# Dispersion of Long and Isolated Single-Wall Carbon Nanotubes by Using a Hydrodynamic Cavitation Method

**DOI:** 10.3390/ma16020466

**Published:** 2023-01-04

**Authors:** Shunjiro Fujii, Shin-ichi Honda, Yoshihiro Oka, Yuki Kuwahara, Takeshi Saito

**Affiliations:** 1 Graduate School of Engineering, University of Hyogo, 2176, Syosya, Himeji 671-2280, Hyogo, Japan; 2 National Institute of Advanced Industrial Science and Technology, 1-1-1, Higashi, Tsukuba 305-8565, Ibaraki, Japan

**Keywords:** nanocarbon materials, single-wall carbon nanotubes, dispersion, cavitation method

## Abstract

Single-wall carbon nanotubes (SWCNTs) are promising materials for electronic applications, such as transparent electrodes and thin-film transistors. However, the dispersion of isolated SWCNTs into solvents remains an important issue for their practical applications. SWCNTs are commonly dispersed in solvents via ultrasonication. However, ultrasonication damages SWCNTs, forming defects and cutting them into short pieces, which significantly degrade their electrical and mechanical properties. Herein, we demonstrate a novel approach toward the large-scale dispersion of long and isolated SWCNTs by using hydrodynamic cavitation. Considering the results of atomic force microscopy and dynamic light-scattering measurements, the average length of the SWCNTs dispersed via the hydrodynamic cavitation method is larger than that of the SWCNTs dispersed by using an ultrasonic homogenizer.

## 1. Introduction

Single-wall carbon nanotubes (SWCNTs) have a wide range of promising applications in electronic devices such as transparent electrodes and field-effect transistors [1]. In particular, thin-film transistors that use SWCNT networks [2,3,4,5] have the potential to be applied in a wide range of electronic devices such as electronic paper and wearable computing systems, and the realization of these electronic devices seems to be difficult for conventional amorphous silicon or polysilicon. Transistors of SWCNT networks can be prepared by the wet process by using the dispersion solution of isolated SWCNTs. However, the dispersion of isolated SWCNTs into solvents remains an important issue for their practical applications. So far, ultrasonication techniques have usually been employed to prepare SWCNT dispersions. Horn-type ultrasonic homogenizers have been widely adopted, and well-dispersed isolated SWCNTs in solvents have been realized. Ultrasonic homogenization is a batch process, in which a rapidly vibrating horn, inserted into a solvent with SWCNTs transfers the ultrasonic energy to them. Therefore, SWCNTs in close proximity to the horn are blasted with large amounts of energy. A high impact force of ultrasonication might be applied to the agglomerates of SWCNTs to obtain an isolated SWCNT dispersion. However, ultrasonication might damage SWCNTs, forming defects and cutting them into short pieces [4,6], which significantly degrade their electrical and mechanical properties. Alternatively, a hydrodynamic cavitation was recently investigated and utilized for various applications, such as the preparation of stable submicron emulsions [7], exfoliation and dispersion of multi-wall carbon nanotube [8], graphene [9], and other two-dimensional materials [10], and dispersion and separation of SWCNTs in organic solvents [11,12], including toluene. In this method, the pressure variations in the flow owing to the unique geometry of the system generate cavitation microbubbles inside a solvent [13,14,15]. Because the hydrodynamic cavitation produces cavitation bubbles more efficiently than conventional ultrasonication techniques [15], a mild dispersion of SWCNTs into the solvent can be achieved, without the reduction of the length of SWCNTs. Additionally, the hydrodynamic cavitation has the merits of full scalability and prevents the incorporation of metal debris that is frequently problematic in the horn-type ultrasonic homogenizer.

Herein, for the first time, a hydrodynamic cavitation dispersion (HCD) method is applied to obtain the dispersion of SWCNTs in water with surfactant. The lengths of SWCNTs dispersed by using HCD method and a well-established ultrasonic homogenizer were compared. It is shown that the average length of SWCNTs dispersed via HCD method was larger than those dispersed by using the ultrasonic homogenizer. The result supports the advantage of HCD method for the retention of the superior electronic properties of SWCNTs during their dispersing process.

## 2. Materials and Methods

**Figure 1:**
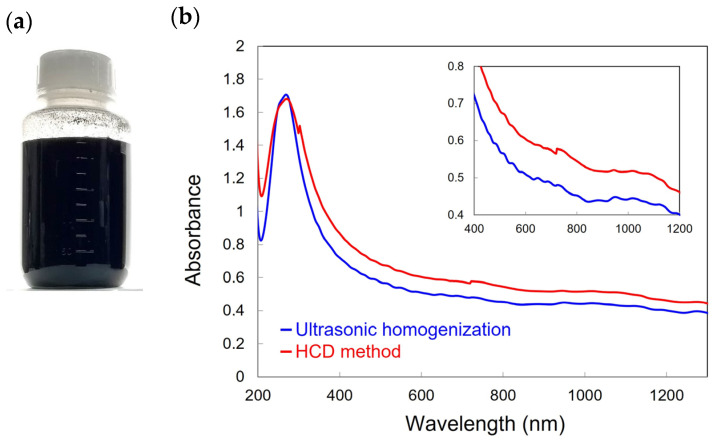
(**a**) Photograph of SWCNT solution dispersed via the HCD method and (**b**) optical absorption spectra of SWCNT solutions after ultracentrifugation. The inset shows enlarged spectra at the visible and NIR range (400–1200 nm).

Herein, SWCNTs produced via an enhanced direct-injection pyrolytic synthesis (eDIPS) method [16,17] were used. Approximately 250 mg of SWCNTs were dispersed in 250 mL of ion-exchanged water with 0.5 wt.% sodium cholate hydrate (SC, Sigma-Aldrich, St. Louis, MO, USA) for 30 min by using an HCD dispersion device (JET PASTER JPSS-X, Nihon Spindle Mfg. Co., Ltd., Hyogo, Japan). Cavitation bubbles were generated by the formation of partial vacuums in water by a swiftly moving rotor. The rotation speed of the rotor was set to 7200 rpm. As shown in Figure 1a, 250 mL of an SWCNT aqueous solution (0.5 wt.% SC) is obtained at a time. Thereafter, the dispersion was ultracentrifuged (Himac CS120FNX, Hitachi, Tokyo, Japan) for 1 h at 100,000× *g* to remove SWCNT bundles, and the resulting supernatant was collected as the SWCNT dispersion. The control sample of SWCNT dispersion was prepared by using the same procedure, except for the dispersion process that uses a horn-type ultrasonic homogenizer (Sonifier 450, Branson, Danbury, CT, USA, output 10%, 30 min). For these SWCNT solutions, the absorption spectra of the SWCNT solutions were measured by using a UV-vis-NIR spectrophotometer (UV-3600 Plus, Shimadzu, Kyoto, Japan). The morphology of the SWCNTs, such as the lengths and diameters, was characterized via atomic force microscopy (AFM) (Dimension Icon, Bruker AXS K.K., Karlsruhe, Germany). The samples for AFM observation were prepared by drop-casting of SWCNT dispersions onto SiO_2_/Si substrates. The AFM images were obtained in air. Dynamic light scattering (DLS) (nanoSAQLA, Otsuka Electronics, Osaka, Japan) measurements for the SWCNT dispersions were performed at a laser wavelength of 660 nm.

## 3. Results and Discussion

Figure 1b shows the optical absorption spectra of SWCNT aqueous solutions (0.5 wt.% SC) dispersed via the HCD method and the ultrasonic homogenization. It is shown that the spectroscopic difference between the ultrasonic homogenization and HCD methods is negligible. This result indicates that a significant dispersion of the isolated SWCNTs can be obtained by using both methods. No distinct peak is observed in the spectra owing to the broad diameter distribution of used SWCNTs [18].

**Figure 2:**
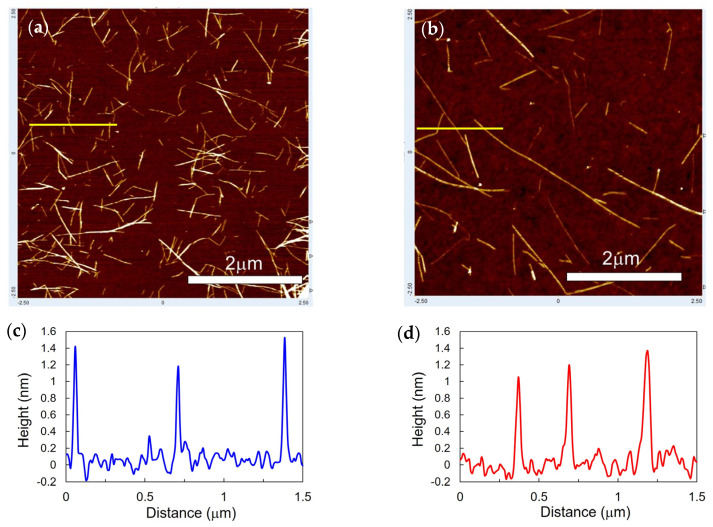
(**a**,**b**) AFM images SWCNTs on SiO_2_/Si, and (**c**,**d**) the height profiles for yellow lines specified in the images. SWCNTs were dispersed by using (**a**,**c**) ultrasonic homogenization and (**b**,**d**) HCD method.

**Figure 3:**
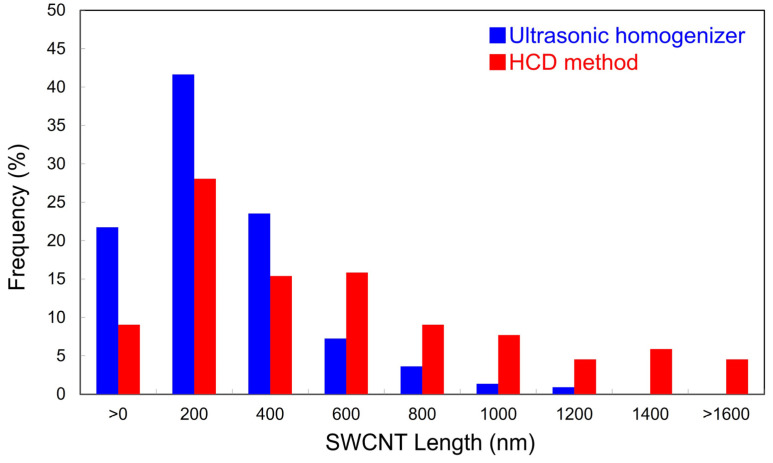
Histogram showing the length distributions of SWCNTs dispersed by using the ultrasonic homogenization (blue) and HCD method (red) with the class interval of 200 nm.

Figure 2a,b show the typical AFM images of SWCNTs dispersed by using the ultrasonic homogenizer and HCD method, respectively. The height profile analysis for AFM images (Figure 2c,d) confirms that most SWCNTs are unbundled. Therefore, we assumed that the characterized length is not for SWCNT bundles but is for isolated SWCNTs. The AFM images clearly suggest retaining the longer length of SWCNTs during the dispersion process of HCD compared with ultrasonication. The distributions of SWCNT length and the average lengths were analyzed from AFM images as shown in Figure 3 and Table 1, respectively, that support the above suggestion. That is, whereas the ultrasonic dispersing results in the relatively short average length of 0.38 μm, the HCD process leads to 0.69 μm length, which is approximately double. By analyzing in detail, the percentage of SWCNTs longer than 1 μm, that is, micrometer-long SWCNTs, was calculated based on the counting number (Table 1). The percentage of micrometer-long SWCNTs was 23% for the HCD method, that is 10 times higher than that obtained for the ultrasonic dispersing.

**Figure 4:**
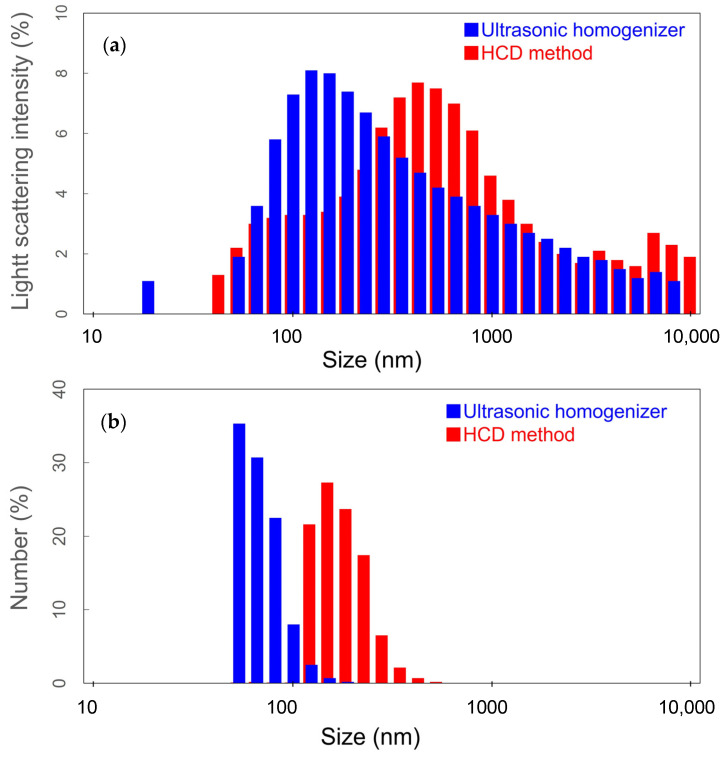
(**a**) Light-scattering intensity and (**b**) particle size distributions in the SWCNT dispersion prepared by ultrasonic homogenizer (blue) and HCD method(red).

We also measured the particle size for SWCNT dispersions by DLS [19] with a 660-nm laser. It should be mentioned that the particle size in the case of SWCNTs would not directly correspond to the tube length, but the sphere-equivalent diameter. During the DLS measurements, the dispersion medium was assumed to be pure water because of the low concentration of dispersant used. Figure 4a shows the light-scattering intensity distributions for the SWCNT dispersion prepared by using the ultrasonic homogenizer and HCD method. By using these results, the light-scattering intensity distributions can be converted into the particle size distributions, as shown in Figure 4b. The distribution shows that the particle size of HCD is several times larger than that of ultrasonication. Considering the similarity between the particle size distribution obtained by DLS and the SWCNT length distribution from AFM analysis, these results would suggest that the particle size distribution presumably correlates with the length distribution of SWCNTs in their dispersion. Therefore, it can be concluded that the DLS results also support the length of SWCNTs dispersed by HCD is larger than the length obtained by ultrasonication dispersing.

As mentioned above, the HCD method provides well-dispersed, longer SWCNTs than ultrasonic homogenization. Here, we discuss the plausible reasons for the higher length values of SWCNTs during the HCD process than the ultrasonication dispersing. During the HCD process, the mild force generated by the expansion and shrinkage of the cavitation microbubbles [15] and the shear force exerted by the rotating impeller can break the agglomerated and bundled SWCNTs into isolated and dispersed components. The dispersed SWCNTs do not contain any bubbles because the cavitation microbubbles disappear under ambient pressure conditions. Therefore, the HCD method has a significant advantage of mild processing, resulting in minimal damage to the SWCNTs. This strategy provides a promising opportunity for the mass production of long and isolated SWCNT aqueous solutions. SWCNTs dispersed via the HCD method are expected to be applied in electronic devices, such as transparent electrodes and thin-film transistors. Furthermore, it is expected that thin-film devices fabricated by using SWCNTs dispersed via the HCD method can exhibit improved performance.

## 4. Conclusions

Herein, the HCD method was applied for the large-scale dispersion of SWCNTs in an aqueous surfactant solution. Based on AFM analysis, it was concluded that the average length of the SWCNTs dispersed via the HCD method is about two times larger than that dispersed by using the ultrasonic homogenizer, and that the large-scale dispersion of long and isolated SWCNTs can be achieved by using the HCD method. The results were also supported by DLS measurements for the SWCNT dispersion solutions. The HCD method has the potential to contribute to the mass production of long-SWCNT dispersions in water containing surfactants.

## Figures and Tables

**Table 1:** Effect of the dispersion method on the length and micrometer-long SWCNT content of dispersed SWCNTs.

Dispersion Method	Average Length (μm)	Percentage of SWCNTs with Length >1 μm
Ultrasonic homogenization	0.38	2%
HCD	0.69	23%

## Data Availability

Not applicable.
